# The interplay of leadership dynamics and person-centred practice in nursing homes: a mixed methods systematic review

**DOI:** 10.3389/frhs.2025.1535414

**Published:** 2025-07-14

**Authors:** Camilla Anker-Hansen, Liv Berit Olsen, Vigdis Abrahamsen Grøndahl, Ann-Chatrin Linqvist Leonardsen, Ann Karin Helgesen, Carina Bååth, Liv Halvorsrud, Gea Restad, Brendan McCormack, Ingrid Femdal

**Affiliations:** ^1^ Department of Nursing, Health and Bioengineering, Faculty of Health, Welfare and Organization, Østfold University College, Halden, Norway; ^2^ Faculty of Health, Science and Technology, Karlstad University, Karlstad, Sweden; ^3^ Faculty of Health Science, Oslo Metropolitan University, Oslo, Norway; ^4^ Susan Wakil School of Nursing and Midwifery, Faculty of Medicine and Health, The University of Sydney, Sydney, NSW, Australia

**Keywords:** attitudes, empowerment, leadership, management, nursing homes, older people, person-centered, role modeling

## Abstract

**Background:**

Implementing a person-centered approach in nursing homes can significantly improve patient satisfaction and care quality while also enhancing job satisfaction among healthcare staff. Leaders play a pivotal role in establishing and nurturing a culture that supports person-centered practices. While there is some empirical evidence, a more comprehensive understanding of how leaders effectively foster and sustain person-centered practices in nursing homes is needed.

**Aim:**

To investigate the role of leaders in fostering person-centeredness within nursing homes.

**Methods:**

The study is based on the PRISMA reporting guidelines. Comprehensive searches were performed in CINAHL and PubMed, with article screening and selection facilitated by Rayyan software. A convergent integrated approach from the Joanna Briggs Institute (JBI) was used to synthesize findings from both qualitative and quantitative studies.

**Results:**

The review included ten studies, comprising six qualitative and four quantitative studies. The results indicate that leadership in nursing homes that fosters person-centeredness involves creating and communicating a shared vision, empowering staff, and ensuring systematic and consistent approaches. Additionally, leaders must embody person-centered values through role modeling.

**Conclusions:**

This systematic review highlights the critical role of leadership in fostering and sustaining person-centered practices in nursing homes. Leaders carry a substantial burden of responsibility. The results suggest that a shift towards a more integrated leadership approach, incorporating both distributed and person-centered leadership models, could promote a more sustainable and supportive environment for both leaders and staff, ultimately enhancing the quality of care. These insights provide valuable guidance for nursing home leaders and policymakers aiming to strengthen person-centered practice.

## 1 Introduction

In nursing homes, a significant majority of residents are frail and vulnerable and cope with multiple health conditions (1). For this study, the term “nursing home” refers to residential care facilities that provide long-term care for individuals who are unable to live independently due to physical or cognitive limitations. These facilities offer a range of services, including assistance with activities of daily living, medical care, and rehabilitation. In some countries, such facilities may be referred to by other terms, such as “care homes,” “residential care facilities,” or “assisted living,” depending on the context and specific services provided.

Despite continuous efforts to improve care quality in nursing homes, a concerning number of residents still face poor care experiences (2). Rosemond et al. (3) suggest that adopting a person-centered approach, which emphasizes residents' relationships, life histories, abilities, and preferences, can be a transformative step in nursing home care. Person-centeredness is often hailed as the “gold standard” of care (4) and has become a cornerstone of healthcare, aiming for high-quality service (5, 6). Person-centeredness can be defined as follows:

“An approach to practice established through the formation and fostering of healthful relationships between all care providers, care receivers, and others significant to them in their lives. It is underpinned by values of respect for persons (personhood), individual right to self-determination, mutual respect, and understanding. It is enabled by cultures of empowerment that foster continuous approaches to practice development.” (5, p. 3)

We have chosen to adopt McCormack and McCance's (5) definition of person-centeredness, as it is recognized as a well-established mid-range theory with a solid empirical foundation (5). This definition is widely applied in academic research and practical implementations of person-centered practice (7–9), making it particularly relevant to our study.

The emphasis on person-centeredness represents a shift towards inclusivity and equality in the professional-patient relationship, aiming to address each person's unique needs. McCormack and Skatvedt (10) outlined four fundamental principles of person-centered practice: treating each person as a unique individual, respecting their rights, establishing mutual trust and understanding, and nurturing collaborative relationships. Person-centered practice encompasses the intricate nature of nursing and the broader healthcare context, emphasizing the significance of all individuals within the healthcare environment. Person-centered practice shifts from the dominant practice focus on “doing” to one of “being”, emphasizing the role of individuals working in healthcare and the significance of relationships with others (11). Person-centered care (PCC) is widely acknowledged as essential for ensuring both the quality of care and quality of life in long-term care settings (12). Research indicates that PCC leads to improved patient outcomes, more efficient resource utilization, reduced costs, and heightened satisfaction among both patients and staff (13). However, person-centered interactions can be challenging as nursing home routines sometimes take precedence over individual needs (8).

Leadership in nursing homes plays a crucial role in shaping staff interactions, the work environment, and the quality of resident care (14–16). Nursing home leaders also play a vital role in ensuring residents receive PCC (15). Over time, various leadership styles have emerged, including distributed, transactional, laissez-faire, transformational, and situational (16). While relational and transformative leadership styles have been identified as the most effective in nursing homes (14), research indicates that passive-avoidant leadership remains the most prevalent (17). Often considered a subtype of laissez-faire leadership, passive-avoidant leadership is marked by disengagement from both tasks and personnel, neglect of staff needs, and inaction in the face of emerging issues. It is frequently described as an absence of active or effective leadership (18) and has been linked to reduced satisfaction with leadership, increased incidence of workplace bullying, and higher levels of absenteeism (19). This leadership style may contribute to a disengaged work culture in which staff feel unsupported, ultimately compromising the delivery of PCC and negatively affecting the well-being of both residents and employees. Given the complex and relational demands of nursing home environments, these outcomes underscore the urgent need to adopt leadership models that are proactive, engaged, and aligned with person-centered values.

In recent years, there has been a growing interest in leadership approaches grounded in person-centered values (20, 21). One such approach is person-centered leadership, described by Eide and Cardiff (22) as “leadership supporting, creating, and securing person-centered values and practices” (p. 96). While closely aligned with the values underpinning the Person-Centered Practice Framework (5), person-centered leadership is not formally included in the framework but offers a complementary perspective on how leadership can foster a person-centered culture in healthcare organizations.

Much of the existing research has focused on associations between specific leadership styles and care outcomes. However, recent studies have underscored the need to consider both leadership behaviors and styles when evaluating the quality of care in nursing homes (23, 24), highlighting the importance of leadership approaches that are collaborative, value-driven, and relational in nature.

One such approach is distributed leadership, which has gained increasing relevance in healthcare settings, particularly where complex care processes require shared and relational leadership practices. Unlike traditional models centered on a single leader, distributed leadership involves the collective enactment of leadership tasks across multiple actors. Leadership is understood not as the responsibility of one person, but as a set of behaviors and interactions embedded within everyday relationships (25, 26). By enabling joint responsibility and shared decision-making, distributed leadership supports core person-centered principles such as empowerment, cooperation, and mutual respect. Evidence from a systematic review indicates that distributed leadership can enhance organizational performance (27), suggesting that this model may also contribute to the development of person-centered cultures in nursing homes.

The management of nursing homes requires systems and processes for planning, implementing, evaluating, and adjusting healthcare delivery in line with national laws and guidelines (28, 29). While these systems demand efficiency and compliance, leaders must also foster principles of compassion, individual attention, and relationship-building. Leadership, particularly when supported through facilitation, plays a vital role in strengthening team collaboration and refining person-centered strategies (20). More broadly, leaders carry both the opportunity and responsibility to shape, nurture, and sustain the cultural ethos of their organizations (30, 31).

However, transitioning to a person-centered approach in nursing homes represents a complex and far-reaching organizational shift (3). Despite growing interest in leadership approaches aligned with person-centered values, there remains limited guidance on how to educate and support leaders in this transformation (21). To date, no systematic review has examined the nuanced leadership dynamics that underpin the facilitation of person-centered practice in nursing homes.

## 2 Methods

This systematic review was conducted to investigate the role of leaders in fostering person-centeredness in nursing homes. The review specifically addressed the following research question:

What are the underlying leadership dynamics that facilitate person-centered practice in nursing homes?

Leaders are defined as individuals holding formal leadership roles in nursing homes, such as nursing home managers and head nurses.

The Joanna Briggs Institute (JBI) Manual for Evidence Synthesis guided the conduct and synthesis of this review (32). The *a priori* protocol was registered in PROSPERO, with the registration number CRD42022366678.

### 2.1 Search strategy

Systematic searches were conducted in the databases CINAHL and PubMed. A specialist librarian was consulted during the development of the search strategy and carried out the searches to ensure rigor. Keywords and MeSH terms were used in various combinations with Boolean operators. The search included terms related to:

• Leadership (e.g., leader*, situational leadership, attitude of health personnel, staff attitude)
• Person-centred care (e.g., person-cent* care, personhood, individualized care, patient-centered care, personalized care, person-directed care planning, person-centred practice framework)
• Care settings (e.g., nursing home*, long-term care, residential facilities, homes for the aged, municipal home*, assisted living)
• Implementation and organizational context (e.g., implementation, culture change, quality improvement, organizational change, innovation, experience*, perspective*, framework)

Full details of the search terms and search strings for each database are provided in Supplementary Material S1.

The study adheres to the PRISMA guidelines for systematic review (33). The inclusion and exclusion criteria are presented in Table 1.

**Table 1:** Inclusion/exclusion criteria.

Inclusion criteria	Exclusion criteria
Peer-reviewed articles	Studies in languages other than English or a Scandinavian language
Studies published between 2012 and 2022	Conference abstracts
Presented data related to how leaders in nursing homes engage in person-centered processes	Review articles
Studies using qualitative/quantitative/mixed methods	Thesis
Reported primary research	Comments
Leader and staff perspectives	Editorials
	Books
	Protocols

**Figure 1:**
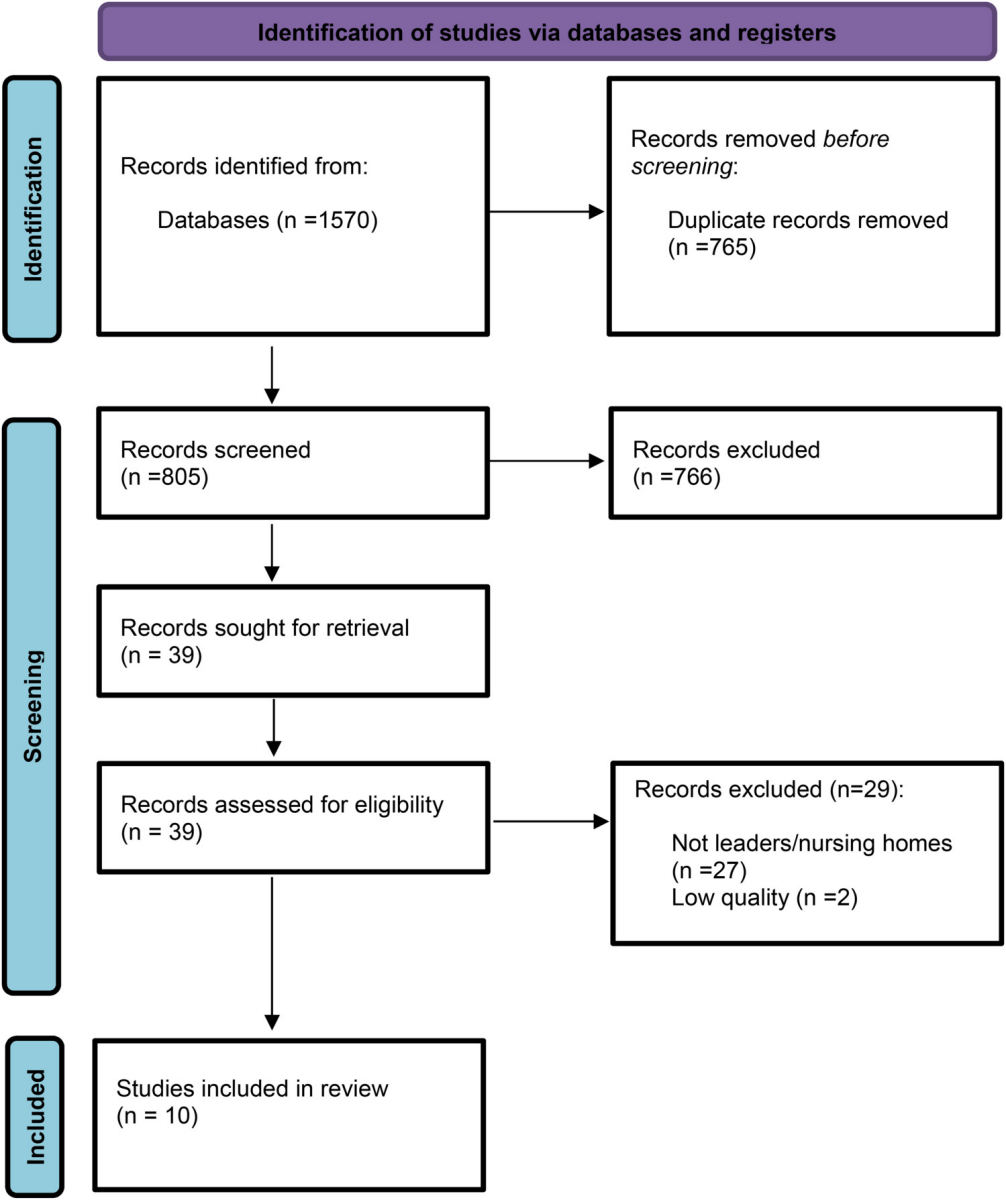
PRISMA 2020 flow diagram for new systematic reviews (33).

The year 2012 was selected as the starting point for the review because healthcare systems have undergone substantial changes over recent decades (34). Studies across diverse healthcare systems with different financial systems are included in this review, as the focus is on leadership dynamics that facilitate the adoption and maintenance of person-centered practice, independent of health policy structures or cultural contexts. We did not restrict inclusion to studies using a specific theoretical framework (e.g., McCormack and McCance's Person-centred Practice Framework). However, studies were only included if they explicitly referred to person-centeredness. Figure 1 presents the PRISMA flow diagram of the study selection process.

### 2.2 Quality assessment of the studies

The quality of the included articles was assessed by two authors (ACLL and CB) using the appropriate JBI Critical Appraisal Tool based on the study design: (i) JBI Critical Appraisal Checklist for Qualitative Research or (ii) JBI Critical Appraisal Checklist for analytical cross-sectional studies. Each author conducted assessments independently and then compared their results. Minor disagreements arose but were resolved through discussion until consensus was achieved. No established parameters exist for weighting qualitative studies (35). In this review, all criteria were deemed of equal importance. A study was classified as high quality if it achieved a score above 70%, moderate quality if it scored between 50% and 70%, and low quality if it scored below 50%, as outlined by Dijkshoorn et al. (36). Only high and moderate-quality studies were included in the final synthesis. Two studies were excluded due to insufficient quality.

### 2.3 Data extraction

The JBI QARI data extraction form for interpretive and critical research (32) served as our tool for data extraction, as outlined in Table 3. Data extraction was conducted for both qualitative and quantitative studies (32, 37). From qualitative studies, we extracted the authors' interpreted findings, such as thematic categories or subthemes, along with supporting interpretations and illustrations. For quantitative studies, we followed the approach described by Lizarondo et al. (37), in which narrative descriptions of results reported by the study authors are extracted and, where appropriate, rephrased or condensed to ensure clarity and relevance to the review objective. This allowed us to integrate quantitative data with qualitative findings by transforming them into textual representations, a process known as qualitization (37). Data extraction was initially conducted by the first author (CAH) and subsequently reviewed by co-authors LBO and IF to ensure accuracy and consistency. No disagreements arose that required further resolution.

### 2.4 Data synthesis

JBI's convergent integrated synthesis approach (32, 37) was used to synthesize data from the included primary studies. The synthesis process is summarized in Table 2, which provides a schematic overview of how the JBI convergent integrated approach was applied. The table outlines the steps from compiling primary data to generating synthesized findings.

**Table 2:** Schematic overview of the synthesis process using JBI's convergent integrated approach (37).

Step	Description
Step 1	Compilation of qualitative findings (authors’ themes, subthemes, and supporting quotations) and qualitized quantitative data.
Step 2	Inductive analysis of the extracted data to develop descriptive categories based on similarity in meaning, involving initial independent coding and discussion.
Step 3	Interpretive synthesis of the categories into overarching synthesized findings through collaborative analysis, ensuring integration across data sources.

**Table 3:** JBI QARI data extraction form for interpretive and critical research.

Study (Ref. number) | Country	Design and method	Study aim	Sample description and setting	Relevant findings
Backman et al. (39) Sweden	A cross-sectional design using valid and reliable questionnaires. Data were analyzed using multiple linear regression, including interaction terms.	To explore the association between leadership behaviors among managers in aged care, and person-centeredness of care and the psychosocial climate.	3,661 staff members in residential aged care facilities in Sweden.	Leadership behavior significantly impacts person-centeredness practice and influences the psychosocial climate. Leadership is most needed in units that are less person-centered, suggesting managers need to lead the way more strongly toward excellence in environments where care is less person-centered. Managers have an important role in promoting, developing, and supporting a PCC philosophy and practice of care.
Backman et al. (40) Sweden	A descriptive interview study with semi-structured interviews.	To explore how managers describe leading towards person-centered care in nursing homes.	Twelve nursing home managers within eleven highly person-centered nursing homes purposively selected from a nationwide survey of nursing homes in Sweden.	Leading towards person-centered care was described as having a personal understanding of the PCC concept and how to translate it into practice and maximising the potential of and providing support to care staff, within a trustful and innovative workplace. Managers coordinate several aspects of care simultaneously, such as facilitating, evaluating, and refining the translation of person-centered philosophy into synchronized care actions. To lead PCC, managers may need to be present at the unit.
Backman (41) Sweden	A national, cross-sectional survey. Descriptive statistics and regression modeling were used to explore associations.	To explore the relationship between leadership, person-centered care, and stress of conscience.	2,985 staff members and their managers from 190 nursing homes throughout Sweden.	Leadership was associated with a higher degree of PCC, indicating that a leadership most prominently characterized by coaching and giving feedback, relying on staff and handling conflicts constructively, experimenting with new ideas, and controlling work individually can contribute to a higher degree of PCC provision. Managers play a crucial leadership role in motivating and empowering staff to deliver PCC.
Duan et al. (42) USA	A cross-sectional design using an online survey.	To (1) test the domain-specific relationships of culture change practices with resident quality of life and family satisfaction, and (2) examine the moderating effect of small-home or household models on these relationships.	102 nursing home administrators in the USA.	Changing restrictive institutions to person-centered homes, referred to as NH culture change, is complex and multifaceted. The findings revealed that culture change operationalized through physical environment transformation, staff empowerment, staff leadership, and end-of-life care was positively associated with at least one domain of resident quality of life and family satisfaction, while staff empowerment had the most extensive effects.
Hamiduzz-aman et al. (43) Australia	Qualitative semi-structured interviews and focus-group interviews.	To explore the factors that shape the dimensions of personalized dementia care in rural nursing homes.	104 Australian care staff participated in interviews and/or focus groups.	The issues of leadership and workplace culture are of importance in the implementation of personalized care in residential dementia care. An authoritative leadership style discourages staff to implement personalized care and to be innovative in dementia care. A lack of consideration of family members’ views by management and staff, together with a poorly integrated, holistic care plan, limited resources, and absence of ongoing education for staff, resulted in the ineffective implementation of personalized dementia care.
Jacobsen et al. (44) Norway	A mixed-method study.	To investigate which factors hindered or facilitated staff awareness related to confidence-building initiatives based on PCC.	299 Norwegian staff members responded to the staff survey at baseline and 228 at follow-up.	The results indicated a development toward more PCC being performed compared to the situation before the education intervention. The involvement of leaders appeared to be a key issue in facilitating successful implementation. Leadership, in interplay with staff culture, was the most important factor hindering or promoting staff awareness related to confidence-building initiatives, based on PCC.
Lynch et al. (45) Ireland	Qualitative approach using a complex action research design with multiple methods: non-participatory observation, critical and reflective dialogues with participants, narratives from residents, focus groups with staff, and reflective field notes	To implement and evaluate the effect of using the PCSLF to develop PCC within nursing homes.	Observation: 11 sessions, Household activity (*n* = 4): 1 leader, 3 staff. Meal times (*n* = 6): 2 leaders, 4 staff. Meal times (*n* = 2): 1 leader, 1 staff. Meaningful interactions (*n* = 5): 1 leader, 4 staff. Leadership behavior (*n* = 7): 7 leaders (across 3 sessions). Connecting with residents (*n* = 5): 1 leader, 4 staff. Team meetings (*n* = 22): 6 leaders, 16 staff (across 2 meetings). Leadership meeting (*n* = 6): 6 leaders. Residents’ Narratives: Convenience sampling at two time points (*n* = 8). 4 residents at time 1, 4 different residents at time 2. Focus Groups (Leaders): Time 2 (*n* = 6): All 6 nursing home leaders. Focus Groups (Staff): Purposive sampling (*n* = 6). 2 staff from each of 3 households (1 nurse, 1 carer per household). All from a private nursing home in Ireland.	Seven core attributes of the leader that facilitate person-centeredness in others were identified relating to the essence of being; harmonising actions with the vision; balancing concern for compliance with concern for person-centeredness; connecting with the other person in the instant; intentionally enthusing the other person to act; listening to the other person with the heart; and unifying through collaboration, appreciation and trust.
Rokstad et al. (46) Norway	Qualitative descriptive design. Focus-group interviews.	To investigate the role of leadership in the implementation of PCC in nursing homes using Dementia Care Mapping.	18 staff members and 7 leaders from 3 different nursing homes in Norway.	The different roles of leadership characterized as “highly professional”, “market orientated” or “traditional”, seemed to influence to what extent the Dementia Care Mapping process led to the successful implementation of PCC.
Røen et al. (47) Norway	Cross-sectional survey.	To explore and understand the association between PCC, and organizational, staff, and unit characteristics in nursing homes.	1,161 Norwegian staff members from 175 nursing homes.	“Empowering leadership” is associated with PCC. Empowering leadership is a managerial style supporting and encouraging the caregivers to take the initiative and to participate in decisions regarding daily care. An innovative climate was associated with PCC.
Røsvik & Mjørud (48) Norway	Qualitative individual interviews.	To explore managers’ and leaders’ experiences to identify factors that facilitate or impede implementation and use of the VIPS practice model in domestic nursing care and long-term care institutions.	17 managers/head nurses representing 10 workplaces in Norway.	Three global categories described the implementation process: factors that impact the decision made at the municipal level to implement PCC, which highlights the decision-making process before the implementation in the unit; requirements for a good start at the unit level, that is leadership commitment, stability among the staff group and staff training; and finally, factors that help to support the new routines in the unit, such as a determined head nurse, leaders who establish structure, mastery, and positive results and supervising the staff.

Thematic categories were developed through an inductive analysis of the extracted data, which included authors' interpretive themes, subthemes, and supporting quotations from qualitative studies, as well as qualitized narrative findings from quantitative studies. During the process, findings were grouped based on similarity in meaning, with attention to recurring concepts, language, and underlying assumptions about leadership and person-centeredness. Contrasting perspectives were also explored to ensure a nuanced interpretation. Initial coding and categorization were performed independently by three reviewers (CAH, IF, LBO), followed by collaborative discussion to refine and consolidate categories.

Subsequently, one researcher (CAH) led the synthesis process by analyzing the descriptive categories for overarching patterns and integrating them into synthesized findings. This interpretive synthesis was carried out in ongoing dialogue with the co-authors (IF and LBO), ensuring that the final themes were grounded in the evidence and represented both convergence and variation across included studies.

## 3 Results

### 3.1 Study selection

The search yielded 1,570 potentially relevant papers. The papers were imported into EndNote software and subsequently transferred to Rayyan (38) for deduplication. Five members of the review author team independently screened the studies by title and abstract (CAH, LBO, LH, AKH, IF). For a paper to be considered relevant, it needed to include the terms “management” and “nursing home,” or their synonyms, in the text, in addition to adhering to the inclusion and exclusion criteria. The reviewers then divided into two groups and compared their results, identifying 39 articles. In the following screening phase, the five review authors independently assessed the full text of the 38 articles for final inclusion. Any discrepancies in selection during the review process were resolved through discussion.

The final sample comprised ten studies: six qualitative and four quantitative (Figure 1). Four of the studies were conducted in Norway, three in Sweden, one in Australia, one in Ireland, and one in the USA. Perspectives from both formal leaders (nursing home managers and head nurses) and non-managerial staff (e.g., registered nurses, enrolled nurses, and nursing assistants) were represented. The quality assessment of the included studies is presented in Table 4.

**Table 4:** Result of the quality assessment of the included studies.

3A.) JBI critical appraisal checklist for qualitative studies		
Study	Criteria^a^ 1 2 3 4 5 6 7 8 9 10	Total/10
Backmann et al. (40)	+ + + + + − − + + +	8/10
Hamiduzzaman et al. (43)	+ + + + + − − + + +	8/10
Jacobsen et al. (44)	+ + + + + − + + + +	9/10
Lynch et al. (45)	+ + + + + − − + + +	8/10
Rokstad et al. (46)	+ + + + + + + − + −	8/10
Røsvik &Mjørud (48)	+ + + + + − − + + +	8/10

^a^ (1) Congruity between stated philosophical perspective and the research methodology. (2) Congruity between research methodology and research question. (3) Congruity between research methodology and methods for collecting data. (4) Congruity between research methodology and the representation of the analysis. (5). Congruity between research methodology and the interpretation of results. (6) Statement locating the researcher culturally or theoretically. (7) Influence of the researcher on the research and vice-versa addressed (8). Participants and their voices adequately represented (9). Is the research ethical according to current criteria or, evidence of ethical approval by an appropriate body. (10) The conclusion drawn from the analysis or interpretation of the data.

**Table:** 

3B.) JBI critical appraisal checklist for analytical cross-sectional studies		
Study	Criteria^a^ 1 2 3 4 5 6 7 8	Total/8
Backman et al. (41)	− + + + + + + +	7/8
Backman et al. (39)	+ + + + + + + +	8/8
Duan et al. (42)	+ + + + + + + +	8/8
Røen et al. (47)	+ + + + + + + +	8/8

^a^ (1) Inclusion criteria clearly defined. (2) Study subjects and setting described in detail. (3) Exposure measured in a valid and reliable way. (4) Objective, standard criteria used for measurement of the condition. (5) Confounding factors identified. (6) Strategies to deal with confounding factors stated. (7) Outcomes measured in a valid and reliable way. (8) Appropriate statistical analysis used.

### 3.2 Result of data synthesis

The data synthesis led to three synthesized findings: (i) Visionary leadership and empowerment; (ii) Consistent and systematic approach for person-centered outcomes; and (iii) Leadership through role modeling. These findings were arrived at through the use of the JBI's convergent integrated synthesis approach, as described earlier (32). Table 5 presents the results of the data synthesis following the convergent integrated approach (37), in which findings from included studies were grouped into thematic categories and further integrated into three overarching synthesized findings. The structure aims to illustrate how multiple qualitative findings were converged through interpretive analysis into higher-order syntheses, supported by excerpts from the primary studies.

**Table 5:** Results

Synthesized finding	Visionary leadership and empowerment	Consistent and systematic approach for person-centered outcomes	Leadership through role modeling
Category	• Importance of clear visions and values • Empowering and enhancing staff performance	• Capability to organize and establish structure systematically • Continuous focus on person-centered outcomes	• Actively participating in care provision • Promoting a safe, supportive environment with a culture of continuous growth
Findings (Excerpts from included studies)^1^	The findings underline the need for a clear and coherent vision to obtain professional development and person-centered dementia care. Transformational and situational leadership, along with a clear vision defining PCC, seemed to be vital for successfully implementing PCC. Leaders have a central role in drawing up a clear and consistent professional vision. The leadership seemed to influence the nursing staff's experiences of empowerment and their ability to put the idea of PCC into action to meet the patients’ needs. Encouraging the staff as a group to be actively involved and take shared responsibility for the residents’ care is crucial, as demonstrated at the ‘highly professional’ nursing home. The staff felt empowered and trusted to make their own decisions in their daily care practice (46). The leaders described having a personal understanding and knowledge of the principles of PCC. The leaders described having a clear vision of what they wanted PCC to be, and how to integrate their vision into practice. Most managers described that talking about what person-centered care is and what it is not and having full focus on the care of the resident, was important. The managers worked actively to concretize the person-centered philosophy and to operationalize this in practice. The person-centered vision was made explicitly exemplifying and verbalising important concepts of PCC. The leaders encouraged the staff to adopt a reflective mindset. Value based issues and dilemmas were solved by turning the focus back to the resident (40). Higher levels of PCC was associated with empowering leadership, among other factors. An innovative climate was associated with PCC, describing this as taking the initiative and encouraging staff to find alternative ways to do things. The finding in the present study shows that especially “empowering leadership” is associated with PCC (47). The result of this study also empirically supports the theory of person-centered nursing confirming that leadership is a prerequisite for PCC on the unit. The impact of leadership behaviors on the psychosocial climate seemed to depend on the level of person-centeredness of care, indicating that leadership behaviors are of utmost importance for the psychosocial climate of staff and residents when the levels of person-centeredness of care are very low. On the other hand, when the person-centeredness of care is low, clinical leadership becomes more important for the overall climate, suggesting that managers need to lead the way more strongly toward excellence in environments where care is less person-centered (39). PCC was described as the organization's ethos, and improving the quality of care was the most important incentive for implementing PCCfor the leaders irrespective of management level. In the VPM, the head nurse is expected to attend each consensus meeting, supervise the staff, ensure the professional standards of the decisions, and provide recognition to the frontline staff. Doing all of this was described as difficult to accomplish but necessary (48). Staff empowerment had the most extensive benefits on resident quality of life, ranging from promoting residents’ positive experience with meal services and day-to-day care to improving psychosocial well-being (specifically dignity, autonomy, and meaningful activities (42). This study illuminates some additional factors that shape the personalized dementia care dimensions, for example, quality of care is impacted by leadership, person-centered communication of staff with residents, and the personal and social life of staff (43). The quantitative data (…) indicates that a positive staff evaluation of their leaders predicts a more positive perception of their institution as to the commitment to PCC (44). The residents who live in Household B have a great life here and our mission is to work as a team to make this vision a reality. the leader needs to be vibrant, have amazing energy to support the team, engender trust and lead on PCC… (Pat, carer in focus group with staff; time 2). The recent team building sessions have strengthened each team member's contribution to the overall team and their belief in the vision… (Mary, carer in focus group with staff; time 2). (…) they are an enthusiastic, flexible and confident team and both leaders of the household work well together showing trust and appreciation for each other and for the overall team… (reflective dialogue with Bell, care manager; time 3) (45).	The leaders described being embedded in PCC in all their day-to-day activities. Leading PCC involves being able to maximize the potential of the team. The leaders reminded the staff about the objectives and goals connected with a approach. These managers also expressed that they wanted to see the person-centered philosophy integrated in all aspects of care and expressed that care routines were also re-directed from intuitional-like care to person-centered care. Assessing and calibrating the extent that staff was integrating PCC into practice was described as important since PCC was perceived as somewhat difficult to maintain. A PCC approach could easily fall off the track, they had to work actively to steer back on track, and it was necessary to hold on and not let go. The leaders applied innovative solutions when organizing work to adapt the organization to the needs and requests of the residents. The managers described the importance of clarifying different team roles and positions of their staff for enhancing PCC. By knowing the individuals in the staff group, the managers could identify different roles in the group and designate different positions so that the group's combined qualities and competencies were utilized to promote person-centered care. The managers explained that identifying and utilizing their staff's unique areas of knowledge and skills enabled the possibility of creating different areas of responsibility for the staff, making it possible for staff to share their skills amongst the team members and residents. The managers explained that identifying relational competencies between staff and residents was central to building and enhancing person-centered relationships. An important aspect for the leaders was to optimize person-centered support structures. Having a clear structure for care planning, as well as routines for evaluating PCC was described as important for development and maintenance, and new forums were developed, and existing forums were optimized and changed to facilitate this. The managers described that they created new forums to lead staff toward engaging in PCC. For example, some managers used existing quality registers for nursing interventions or as baseline tools to evaluate initiatives. The leaders organized and attended care meetings, and being involved in creating care plans based on the residents’ needs provided a clear structure to follow. The managers described changing existing forums to facilitate PCC. For example, it was described that ordinary workplace meetings were used as forums to raise person-centered issues, as well as to follow-up on person-centered interventions (40). Stability in the unit was necessary in order to develop the competence and skills required to execute the functions of the VIPS practice model: [It is important] that the turnover is low, that people know what the primary tasks are, that they can document things, simply a well-driven unit. You need to sort out any chaos before you can implement something that requires professionalism and structure because you need structure to make it work. Upholding the new routines for the consensus meeting was highly dependent on the head nurses. In fact, their engagement was described as pivotal: It is the head nurse who makes the difference . . . a leader who schedules the meetings and organizes the time to hold them. The new systematic way of working also meant that interventions should be adjusted if necessary. The head nurse reminded the staff to be alert and make observations: I tell the frontline staff ‘You need to document it [how the interventions work], then we can discuss it. You need to observe it and look into it before the consensus meeting when we are evaluating it’. To manage to conduct the consensus meetings regularly in the units, the meetings were all planned ahead regarding time and participants: For things to work, you need leaders who create structure, structure with fixed meeting times, and full-time employees [present]. They made schedules, so staff could be prepared for the meetings: We planned the next meeting early on; they [the frontline staff] knew 14 days in advance. It gives them time to process it in their heads (48). The consistent and systematic pursuit of effectiveness and service was perceived as conflicting with the values of PCC (46). This leader carefully mapped which residents shared things in common with each other and with staff members and carefully planned for the ‘right matching’ and also, for the gradual implementation of the decision-making model. (44). A flamingo looking at its own reflection in the water represents the importance of getting the balance right between compliance and the culture of PCC. It constantly changes… sometimes the ripples make the reflection bigger…when a HIQA inspection is due…compliance seems heavier than person-centeredness…constant emphasis on paperwork. Having a consistent team helps to keep the balance… the images of the flamingo are equal then… (Maggie, staff nurse in focus group with staff; time 2 (45).	The staff felt leaders appreciated, supported, and encouraged their efforts for the residents and felt supported in delivering quality care. Participation from leaders in the nursing practice was considered crucial by the staff. In one of the nursing homes, the leaders were present on the ward daily making the staff feel supported and engaged. In another nursing home, the leaders could not be present at the ward and take part in tasks, which seemed to result in frustrated leaders and resigned staff. The leaders saw themselves as role models for the care staff. Leaders have a central role in being continuously supportive of the care staff and taking an active part in the care practice as role models (46). The impact of leadership behaviors on the psychosocial climate seemed to depend on the level of person-centeredness of care, indicating that leadership behaviors are of utmost importance for the psychosocial climate of staff and residents when the levels of person-centeredness of care are very low (39). Leading PCC involves providing individual support to care staff, within a trustful and innovative atmosphere. The leaders reported by being present in the unit on a daily basis and making own assessments, and taking control of the care situation, if necessary, the extent of PCC delivered was assessed. The leaders reported that they were able to coach staff in nursing interventions and also remind staff of objectives and priorities in conflict situations. Promoting a person-centered atmosphere was described to be important for enabling person-centered being and doing. An atmosphere underpinned by mutual trust, creativity, and innovation was central to providing PCC. An atmosphere of trust was described as crucial for developing PCC. Several managers described that one way of creating trustful relationships was by providing constructive and positive feedback to staff about their performance. Trust was achieved by the validation and recognition of staff competence and gradually handing over responsibility for the person-centered care to staff. The delegation was described to show that trust was in place. The managers described the importance of creating a space that encouraged staff to think outside the box and encouraged chance-taking and testing creative solutions in daily care as person-centered care was considered neither static nor standardised. Most managers described that it was important to be a role model and lead by example by being involved in the care. Also reported that they recognized, highlighted, and confirmed good examples in the clinical practice and used positive situations as a benchmark for care planning, and positive psychology seemed to be an important feature in supporting a person-centered atmosphere. Another important aspect of leading towards person-centered care was described as maximizing person-centered team potential. This was outlined as making the group function as a team, utilizing their positions, as well as competencies was considered necessary for promoting person-centered care (40). This study provides insights that leadership most prominently characterized by behaviors such as experimenting with new ideas, controlling work closely, relying on his/her subordinates, coaching, and giving direct feedback, and handling conflicts in a constructive way is positively associated with less staff stress of conscience as well as with increased PCC. The positive correlation between leadership and PCC suggests that by fostering trust, delegation, and innovation, managers can further promote this care approach (41). Leadership and organizational culture were found by the staff as key to practicing a holistic care management plan for Residents with dementia. The hierarchical leadership and relationships discouraged them to work as a team for incorporating the components of personalized dementia care in their everyday care service. Some staff stipulated that how authoritative leadership influenced their care activities. While some clinical managers discussed the difficulties in engaging the care workers into personalized care, several care workers emphasized the need to improve respect among the staff [horizontal and vertical] in order to implement a new model of care. (43). The respondents underlined that leaders at all levels in the organization had to be committed for the ethos of PCC to become a reality: We were very clear that this was not just another project; this should be the way we work, how we do things (#8). Some staff needed support from the head nurse to do this, and one head nurse said she encouraged them by stating: This is your job, and I know you can do it (#15) (48). Fostering leadership of direct care staff also showed a favorable impact on three quality of life domains including dignity, autonomy, and meaningful activities. (42). Respondents who evaluated their leaders as open and inclusive were most likely to think that their institution is committed to PCC. The leader gave freedom to staff with regard to how they organized their daily tasks, but she immediately intervened when the care work did not work out well. Leadership and staff culture appear to be pivotal factors in promoting or hindering PCC, a necessary pre-condition for confidence building initiatives in staff-patient relationships, based on PCC. Respondents who evaluated their leaders as open and inclusive were most likely to think that their institution is committed to PCC. The leadership stands out as a very important factor. As an example from the facilitator notes, in one home, the number of attendants dropped when the leader was on sick leave, from an average of 13 in the first four sessions, to five in the last 2 months when the leader was absent. How staff perceived their leaders was found to predict how staff perceived the presence or absence of PCC. By acting as internal facilitators, the leaders’ activities directly and indirectly increased the potential for success stories in terms of more person-centered and restraint- free care to happen. The ethnographic studies make clear, however, that the manner in which the leaders are involved is important for the success or lack of success of the implementation (of PCC) (44). She [the leader] treats us all like we all have star qualities—she knows the stage each of us is at. I think she works hard at getting us enthusiastic about doing the best we can …She's always supporting us to develop innovative ways to give care in a person-centered way… (Noleen, staff nurse in focus group with staff; time 2). They identified a resident, and with the resident's agreement, worked together to tailor the resident's shower, breakfast, medications and dressings all around what suited the resident—not as a series of isolated tasks, but in a smooth integrated way. The two leaders brought this change in practice to the monthly household team meeting in order to increase the staff's knowledge and understanding of PCC using the “living” example, and to help integrate the approach into their day-to-day practice. (45).

### 3.3 Visionary leadership and empowerment

The results in this synthesized finding highlight how the leaders had clear and shared visions for person-centered practice through the following categories: (i) Importance of clear visions and values, and (ii) Empowering and enhancing staff performance.

#### 3.3.1 Importance of clear visions and values

Several of the reviewed papers identified the need for a clear, coherent vision in fostering professional development, establishment, and delivery of a person-centered practice (39, 40, 45–47). Leadership did not encompass a passive role; the leaders were at the forefront, actively shaping and defining visions and values into the professional practices of their teams. From the statement, “the managers in this study described having a personal understanding and knowledge of the principles of PCC, and also a clear vision of what they wanted it to be’ (40, p. 175), it is evident that leaders had a deep connection with the principles of PCC. For them, the vision of PCC was not just a mere policy tick-box but resonated with their beliefs and understanding about care. Further, several staff members had high expectations of their leaders in terms of supporting the staff team, as illustrated by the following quote:

“The leader needs to be vibrant, have amazing energy to support the team, engender trust and lead on person-centered care…” Carer (45).

#### 3.3.2 Empowering and enhancing staff performance

Findings in this category focused on how leadership facilitated empowerment, autonomy, and the enhancement of staff performance within the realm of person-centered practice. Four studies highlighted the critical role of leader-facilitated staff empowerment, viewed from various perspectives (42, 45–47). Duan et al. (42) discovered that empowering staff significantly improved the quality of life for residents, with positive impacts observed in meal services, daily care, and psychosocial well-being aspects like dignity, autonomy, and engaging activities. Similar findings were reported by Røen et al. (47) and Rokstad et al. (46), both noting a positive correlation between PCC and empowered staff. Rokstad et al. (46) further observed how leadership seemed to influence staff's sense of empowerment and their ability to implement PCC effectively:

“The staff felt empowered and trusted to make their own decisions in their daily care practice.” Leader. (46, p. 23).

Elevated levels of PCC were associated with empowering leadership, among other factors. An innovative climate, typified by initiative and the encouragement of alternative methods and approaches, was also linked to PCC (47). Lynch et al. (45) found that the leaders empowered staff performance by encouraging innovative, individualized approaches, aligning with each team member's development level.

### 3.4 Consistent and systematic approach for person-centered outcomes

In this synthesized finding, the importance of having a systematic approach and a structured plan in the workplace to achieve person-centered goals is highlighted. In addition, the importance of maintaining focus on the goal was emphasized. The synthesized finding is reflected in the subcategories (i) Capability to organize and establish structure systematically, and (ii) Continuous focus on person-centered outcomes.

#### 3.4.1 Capability to organize and establish structure systematically

Four of the articles emphasized the importance of having a systematic approach to person-centered practice (40, 44, 46, 48). Leaders strategically utilized both new and existing forums to promote person-centeredness. Whether through ordinary workplace meetings or specially created platforms, the agenda often revolved around person-centered issues and interventions. This involved planning meetings, scheduling various activities, and reminding the staff of objectives and goals connected with a person-centered approach (40, 48). Furthermore, a consistent and stable team in the department was underscored as a crucial component in achieving systematic organization and structure (45, 48).

The way of organizing and being systematic in the approach to achieving a person-centered practice also manifested itself in other ways, such as identifying different qualities among the staff so that staff and residents were matched based on the chemistry they had with each other (44), or seeing themselves as a team where the aim was to bring out the best in each other as quoted by this leader:

“I talk a lot about that we are like a football team, everyone cannot be Ibrahimović… but I think it's so important… “I think like this, we must have positions, as we are a team, sometimes you do more of this and less of that, but that does not mean that we are doing a poorer result, maybe better, as the result will be better when we position ourselves.” (40, p.178).

Thus, the capability to organize and establish structure systematically was not just about administrative processes or maintaining consistency within staff. The focus also lay in understanding how to best utilize the unique strengths and dynamics of each staff member to achieve the overarching goal of person-centered practice.

#### 3.4.2 Continuous focus on person-centered outcomes

Within this category, the need for having a continuous focus on person-centeredness became evident (40, 46, 48). Establishing person-centered practice was not a one-time event; rather, it required sustained dedication and vigilance. As one leader articulated:

“We have to keep the idea of person-centeredness warm all the time” (46, p. 21).

Encouraging staff to observe, reflect, and share their thoughts seemed to be emphasized as valuable in the process of enhancing person-centered outcomes. In maintaining focus on person-centered practice, several elements were involved. One of the elements was to keep the awareness high about the concept, and to see and recognize the residents' needs:

“If you lived here, what would be most important for you? - What do you think is most important for the persons living here?” Leader (40., p.176).

Røsvik and Mjørud (48) on their side pointed out the importance of observing and documenting how the interventions worked, in order to evaluate together in staff meetings.

### 3.5 Leadership through role modeling

A recurring topic was the importance of the leaders leading by example. The importance of role modeling was emphasized both by the leaders and by the employees. The synthesized findings are divided into the following categories: (i) Actively participating in care provision, and (ii) Promoting a safe and supportive environment with a culture of continuous growth.

#### 3.5.1 Actively participating in daily routine

When leaders were visibly present and validated the staff's approach to resident care, staff satisfaction and their motivation to provide personalized care were notably enhanced. The role of a leader extended beyond just oversight; they acted as both a support mechanism for the staff and as an integral part of residents' day-to-day care (40, 44–46, 48). As one leader detailed:

“I am out on the wards, I'm visible on a daily basis, and I follow up by asking questions: How is it going? How are we doing? What can we do here? How can we think concerning this…?” (40, p. 177).

Rokstad et al. (46) documented varying perspectives on this theme. In one nursing home, leaders and staff concurred on the importance of leaders' involvement in daily care. The care staff felt both inspired and supported to deliver quality care, and the leaders conveyed appreciation for their dedication and skills. In contrast, another nursing home saw a disconnect when leaders couldn't be present, resulting in disheartened leaders and a resigned staff. Both groups found this scenario challenging, with one leader commenting:

“I cannot be present on the ward on a daily basis, so I have to lead the care practice through others. I find this frustrating.” (46, p. 21).

Another study found that staff in one nursing home faced challenges with a leader who did not engage in daily activities, describing their leader as “distant” and “lacking involvement in staff and residents’ matters.” (44).

This category also shines a light on leaders as role models in a person-centered practice. Their involvement in nursing was viewed as more than just practical assistance; it symbolized leading by example (40, 43, 46):

“Most managers described that it was important to be a role model and lead by example by being involved in the care.” (40).

The active involvement of leaders in caregiving underscored how a person-centered approach was as much about hands-on participation as about guiding principles.

#### 3.5.2 Promoting a safe, supportive environment with a culture of continuous growth

Several studies highlight the significance of not only adopting a person-centered approach for residents but also treating staff according to the principles of person-centeredness (40, 41, 43, 44, 46, 48). Moreover, it seemed like when employees perceived their leaders as open and inclusive, they were more inclined to believe that the institution genuinely valued person-centeredness (44).

Another key element identified was the commitment of leaders to embed the ethos of person-centeredness deeply within the organizational culture (42, 44, 48). The leaders in Røsvik and Mjørud's study (48) emphasized that leaders, regardless of their management level, should prioritize PCC as the main framework for addressing value-based issues and ensuring person-centered solutions for residents:

“The respondents underlined that leaders at all levels in the organization had to be committed for the ethos of person-centered care to become a reality: We were very clear that this was not just another project: this should be the way we work, how we do things.” (48).

Rokstad et al. (46) also emphasized the inherent responsibility of leaders to provide continuous support to care staff. This finding is echoed in Lynch et al. (45), where a nurse described how her leader demonstrated support:

She treats us all like we all have star qualities—she knows the stage each of us is at. I think she works hard at getting us enthusiastic about doing the best we can … (45).

Further findings from Backman et al. (39) illustrated the impact of leadership behaviors, especially in shaping the psychosocial climate for both staff and residents, with this influence being even more pronounced when PCC was inadequate.

## 4 Discussion

The findings from this study highlight several key dynamics underlying effective person-centered leadership in nursing homes, particularly the importance of visionary leadership and empowerment, a consistent and systematic approach, and the importance of modeling person-centered values and behaviors. This discussion aims to interpret the key findings of the study and situate them within the broader context of relevant existing literature.

### 4.1 Visionary leadership and empowerment

The findings underscore the necessity for nursing home leaders to possess a cohesive vision and set of values aligned with person-centered principles, ensuring these visions transcend superficial policies and resonate with leaders' core beliefs (39, 40, 45–48). These findings align with previous research indicating that leaders who deeply understand and embody person-centered principles are better positioned to implement them effectively in practice (30). Earlier studies also support the importance of a shared vision as an essential feature of leadership behavior. Martin et al. (49) found that vision provides orientation and meaning for leaders and their teams, helping them focus their energies and engage in the transformation of practice. A 2022 systematic overview of reviews by Feldthusen et al. (50) describes numerous prerequisites for facilitating person-centered practices in healthcare, including the formation of a vision.

The correlation between empowered staff and person-centeredness underscores the significance of effective leadership in fostering staff empowerment through support, autonomy, and opportunities for agency (45–47). Prior research (51) corroborates these findings, suggesting that empowering staff improves outcomes for nursing home residents and enhances staff motivation and job satisfaction. Additionally, Ta'an et al. (52) found that highly empowered nurses displayed higher performance than less empowered nurses in hospitals. Conversely, Feldthusen et al. (50) found that a lack of influence over policies, procedures, and practices contributed to feelings of disempowerment among healthcare professionals. These factors, coupled with rising workloads and insufficient support, adversely impacted their psychological well-being and their ability to deliver PCC (50).

### 4.2 Consistent and systematic approach for person-centered outcomes

The findings indicate that fostering person-centered practices requires systematic approaches and structured planning from leaders (40, 48). For instance, one nursing home in the study implemented systematic review meetings to evaluate care plans and PCC practices, which were deemed crucial for developing and maintaining person-centered practice (40). This finding aligns with international literature, where previous research supports the necessity of a systematic approach and regular evaluation to sustain high-quality person-centered practice (11, 53). These findings suggest that nursing home leaders should prioritize the development of structured care planning and evaluation routines to ensure consistent and high-quality PCC.

A stable workforce was identified as critical for achieving systematic organization and structure, ensuring a well-coordinated department, and promoting expertise development among staff (48). Stable staffing allows for continuity of care, which is essential for building trust and understanding between residents and caregivers. When staff members are familiar with the residents and their specific needs, they can provide more personalized and effective care (5). However, research by Moore et al. (54) suggests that consistent leadership may be even more critical. Consistent leadership provides direction, stability, and a clear vision, which are vital for sustaining person-centered practices (22). These findings underscore the importance of maintaining a stable workforce and ensuring continuity in leadership roles to effectively implement and maintain person-centered practice.

### 4.3 Leadership through role modeling

A recurring theme was the profound impact of leaders actively modeling person-centered behaviors (40, 43, 45, 46). By serving as visible role models, the leaders reinforced the importance of person-centered values and inspired staff to adopt similar practices. Numerous studies have underscored the leader's role as a model for expected behaviors (49, 54–57). However, what sets this context apart is that leaders also serve as role models in their execution of daily patient care, as evidenced in the study by Rokstad et al. (46), where staff regarded leader participation in nursing practice as crucial. While leader involvement in daily care can enhance understanding and presence, several challenges may emerge. Challenges such as role confusion, time pressure, insufficient clinical competence, and inadequate resource allocation can impede effective leadership and optimal care. Of particular concern is the potential lack of clinical competence among leaders. Although many leaders possess healthcare backgrounds (58), their clinical skills may not be as current as those of staff who work with patients daily. Moreover, Kirchhoff and Karlsson (59) found that first-line nurse managers frequently face role conflict or feel 'squeezed' by the competing demands of their responsibilities as registered nurses and leaders. This dual pressure can result in significant stress, emotional exhaustion, and an inclination to resign from their leadership roles.

A key finding was that the majority of the included studies emphasized the importance of adopting a person-centered approach not only for residents but also for treating staff according to the principles of person-centeredness (40, 41, 43, 44, 46, 48). Such findings illustrate the paradigm shift from the traditionally PCC, which primarily focuses on the patient as the sole important person in the relationship, to person-centered practice, which encompasses all individuals in the relationship, including healthcare professionals (7). Buetow (60) refers to this shift as viewing patients and healthcare personnel as “moral equals,” indicating that to provide effective PCC, healthcare professionals must also feel that their personhood is respected and recognized.

Backman et al. (39) discovered that the influence of leadership behaviors on the psychosocial climate was contingent on the degree of PCC, suggesting that leadership behaviors are critically important for the psychosocial well-being of staff and residents. Furthermore, Jacobsen et al. (44) found that staff perceptions of their leaders were indicators of the presence or absence of PCC in the nursing home. These findings align with research by Seljemo et al. (23) and Zonneveld et al. (24), who emphasize that the significance of leadership behaviors, rather than just leadership styles, is crucial in nursing home care.

### 4.4 Rethinking leadership expectations in nursing homes

The data from all included studies underscore the extensive and multifaceted expectations placed on leaders in nursing homes (39–48). Beyond ensuring the implementation of person-centered practices, leaders are tasked with a wide range of responsibilities, including administrative tasks, role modeling, and direct involvement in care activities (40, 43–46, 48). Persistent challenges in nursing home leadership, such as understaffing, financial constraints, limited resources for staff development, and blurred work-life boundaries, further exacerbate expectations (58, 61, 62). Such demands mirror the traditional “heroic” model of leadership, where leaders are expected to manage and resolve all organizational issues independently (63). This model raises questions about its feasibility and sustainability in the context of modern nursing homes.

There is an apparent contradiction between the expectations placed on leaders and the principles of person-centered practice, which advocate for shared responsibility and collaborative approaches (5). A disconnection between expectations and the support provided to leaders can lead to burnout and reduced effectiveness in leaders (59, 62) and diminish their ability to foster a person-centered culture. This issue highlights the need to rethink traditional leadership models in nursing homes.

### 4.5 Shifting towards integrated leadership models

The findings of this review point to the potential benefits of shifting towards a more integrated leadership model that aligns with person-centered values. In particular, distributed leadership may offer a valuable contribution by supporting a more balanced distribution of responsibilities and tasks across different organizational levels (64). In this model, administrative duties may be delegated to specialized personnel, while clinical leadership is exercised by experienced nurses closer to care delivery. By embedding distributed leadership within broader person-centered strategies, nursing homes may cultivate cultures where leadership is enacted through relationships rather than imposed hierarchically. This can enhance staff engagement and competence (62, 65) and support the sustainable implementation of person-centered practices. Moreover, person-centered leadership plays a crucial role in nurturing such practices by emphasizing staff empowerment, fostering teamwork, and aligning leadership actions with the core values of PCC (22). According to McCormack and McCance (11), the goal of person-centered processes is to create a “healthful culture”, an environment that promotes both staff well-being and quality of care. Emerging research on healthful leadership further reinforces its role in establishing supportive and sustainable workplaces (66).

By integrating the principles of person-centered and distributed leadership, healthcare organizations can enhance the well-being of both staff and leaders, ultimately improving care outcomes (11, 22, 64). Further support for this integrated approach comes from recent work by Cable, McCance, and McCormack (67), who explored how person-centered nursing leadership can be cultivated through transformative professional development. They emphasize that becoming a person-centered leader is a process of *knowing, being, and becoming*, an internal journey that fosters authenticity and relational depth in leadership.

Taken together, these insights suggest that developing integrated leadership models may be key to the sustained success of person-centered practices in nursing homes.

## 5 Conclusion

This systematic review has identified the underlying leadership dynamics facilitating person-centered practice in nursing homes. The analysis revealed three key themes: visionary leadership and empowerment, a consistent and systematic approach to achieving person-centered outcomes, and leadership through role modeling. The findings collectively indicate that substantial responsibility lies with leaders to effectively implement and sustain person-centered practice, in addition to fulfilling their broader managerial duties and obligations. These findings suggest a potential benefit of exploring a more integrated leadership model that draws on distributed and person-centered leadership models. Such a model could lead to a more sustainable and supportive environment for both leaders and staff, ultimately improving the quality of care. This synthesis of existing research provides valuable insights for nursing home leaders and policymakers striving to enhance PCC and highlights the importance of supporting leaders in their efforts to create and sustain person-centered environments.

### 5.1 Strengths and limitations

The strength of the study lies in summarizing knowledge in an area with limited existing evidence. Furthermore, the study is conducted systematically and rigorously, adhering to a recognized framework for systematic reviews. The included studies were critically appraised by multiple reviewers to enhance objectivity and reduce bias.

However, some limitations are evident in this review. The most notable is the imbalance in the distribution of findings among the included articles. Some articles contribute numerous findings, while others provide less. To ensure transparency, the details of which findings are extracted from each article are presented in the results section (see Table 5).

Of the ten included studies, seven were conducted in Norway and Sweden. This raised questions about our search terms and whether different words or concepts might be used in other countries. We extensively used various MeSH terms and examined search terms from comparable studies. Additionally, a specialized librarian conducted the searches. Despite these efforts, we acknowledge the possibility of overlooked factors. Furthermore, the review included only two databases, CINAHL and PubMed. While these databases are highly comprehensive within the scope of nursing and health services research, the use of additional databases might have yielded a small number of additional studies, and this is acknowledged as a limitation.

In our searches, we have not differentiated between professional and administrative leadership, and there might be differences in how closely these various levels work with the staff. There are also different ways of organizing nursing homes in various countries, which have not been considered in this study.

In addition to the limitations already discussed, we acknowledge potential methodological and theoretical constraints in this review. Methodologically, the search was limited to two databases (CINAHL and PubMed), which may have excluded relevant studies indexed elsewhere. Furthermore, while our inclusion criteria focused on studies that involved formal nursing home leaders, the variation in how leadership roles are defined and reported across countries and studies may have introduced some ambiguity.

Variability in study designs, populations, and outcome measures has made drawing definitive conclusions challenging, but such diversity also provides a comprehensive overview of the existing evidence and highlights areas where further research is needed.

### 5.2 Implications of the results for practice, policy, and future research

The findings of this review highlight the need for leadership approaches in nursing homes that are actively aligned with person-centered values and enacted through everyday leadership behaviors. In practice, this calls for leaders who can articulate and embed a clear vision for care, empower staff, and lead by example through consistent engagement in care provision. Establishing such leadership requires not only structural support but also the cultivation of reflective practice, where leaders routinely assess and adapt their approaches based on feedback, values, and situational demands.

From a policy perspective, these findings point to the importance of leadership development programs that prioritize relational and values-based competencies alongside organizational skills. Policies aimed at improving care quality in nursing homes should therefore support leadership models that encourage reflection, staff involvement, and shared responsibility.

Future research should explore how leadership practices can be systematically developed and sustained over time to promote person-centered practice in nursing home settings. Longitudinal studies may help clarify how specific leadership behaviors support the creation of person-centered cultures, enhance staff well-being, and improve person-centered outcomes for residents.

## Footnotes

^1^The findings are presented article by article, and the reference is positioned after the final excerpt from each article.
