# Supplementary material for: The interplay of leadership dynamics and person-centred practice in nursing homes: a mixed methods systematic review
Source: Front Health Serv. 2025 Jul 14;5:1535414. doi: 10.3389/frhs.2025.1535414 (PMC12301321; doi:10.3389/frhs.2025.1535414)
Supplement: Supplementary file 1 [file Table1.docx]

| **keywords**   - leader* - situational leadership | **keywords**   - Attitude of health personnel - Attitude* - Staff attitude | **keywords**   - person-centered practice framework - person-centred care - person-centred healthcare - patient-centered care - people centered care - person-centredness - personhood - **individualized** **care** - person-directed care planning - person centredness - person-centered nursing framework - personalized care - pe-ce | **keywords**   - «homes for the aged» - Long Term Care - municipal home* - Nursing home* - residential facilities - Residental care - «Long-term care facilities for older people» - assisted living | **keywords**  **«health plan implementation»**  **culture change**  **framework**  **quality improvement**  **organizational change**  **program implementation**  Implementation  Perspective*  innovation  experience*  involvement |
| --- | --- | --- | --- | --- |

| 1. Leaders/leadership AND attitudes AND person-centred healthcare (S1 AND S2 AND S3)   1639 hits |
| --- |
| **((((((((((Institutional Management Teams[MeSH Terms]) OR (Leadership[MeSH Terms])) OR (Influentials[MeSH Terms])) OR (Personnel Management[MeSH Terms])) OR (leader*[Title/Abstract])) OR (situational leadership[Title/Abstract])) OR ("Nursing Homes/organization and administration"[Mesh])) OR ("Institutional Management Teams/organization and administration"[Mesh])) OR ("Personnel Management/organization and administration"[Mesh])) AND (((((((((Attitude of Health Personell)[MeSH Terms]) OR (Attitude of Health Personell[Title/Abstract]))) OR (Attitude[MeSH Terms])) OR (Attitude[Title/Abstract])) OR (Work Engagement[MeSH Terms])) OR (Work Engagement[Title/Abstract])) OR ("staff attitude*"[Title/Abstract])) OR ("Attitude of Health Personnel/organization and administration"[Mesh])) OR ("Attitude/organization and administration"[Mesh]))) AND (((((((((((((((((((Patient-Centered Care[MeSH Terms]) OR (Patient-Centered Care[Title/Abstract])) OR (Patient Care Planning[MeSH Terms])) OR (Patient Care Planning[Title/Abstract])) OR ("Patient Care Planning/organization and administration"[Mesh])) OR ("Patient-Centered Care/organization and administration"[Mesh])) OR (person-centered practice[Title/Abstract])) OR (person-centred care[Title/Abstract])) OR (person-centred healthcare[Title/Abstract])) OR (patient-centered care[Title/Abstract])) OR (people centered care[Title/Abstract])) OR (person-centredness[Title/Abstract])) OR (personhood[Title/Abstract])) OR (individualized care[Title/Abstract])) OR (person-directed care planning[Title/Abstract])) OR (person centredness[Title/Abstract])) OR (person-centered nursing framework[Title/Abstract])) OR (personalized care[Title/Abstract])) OR (pe-ce[Title/Abstract]))** |

| 1. Leaders/leadership AND attitudes AND person-centred healthcare and institutions for older people (S1 AND S2 AND S3 AND S4) 246 treff hits |
| --- |
| **(((((((((((Institutional Management Teams[MeSH Terms]) OR (Leadership[MeSH Terms])) OR (Influentials[MeSH Terms])) OR (Personnel Management[MeSH Terms])) OR (leader*[Title/Abstract])) OR (situational leadership[Title/Abstract])) OR ("Nursing Homes/organization and administration"[Mesh])) OR ("Institutional Management Teams/organization and administration"[Mesh])) OR ("Personnel Management/organization and administration"[Mesh])) AND (((((((((Attitude of Health Personell)[MeSH Terms]) OR (Attitude of Health Personell[Title/Abstract]))) OR (Attitude[MeSH Terms])) OR (Attitude[Title/Abstract])) OR (Work Engagement[MeSH Terms])) OR (Work Engagement[Title/Abstract])) OR ("staff attitude*"[Title/Abstract])) OR ("Attitude of Health Personnel/organization and administration"[Mesh])) OR ("Attitude/organization and administration"[Mesh]))) AND (((((((((((((((((((Patient-Centered Care[MeSH Terms]) OR (Patient-Centered Care[Title/Abstract])) OR (Patient Care Planning[MeSH Terms])) OR (Patient Care Planning[Title/Abstract])) OR ("Patient Care Planning/organization and administration"[Mesh])) OR ("Patient-Centered Care/organization and administration"[Mesh])) OR (person-centered practice[Title/Abstract])) OR (person-centred care[Title/Abstract])) OR (person-centred healthcare[Title/Abstract])) OR (patient-centered care[Title/Abstract])) OR (people centered care[Title/Abstract])) OR (person-centredness[Title/Abstract])) OR (personhood[Title/Abstract])) OR (individualized care[Title/Abstract])) OR (person-directed care planning[Title/Abstract])) OR (person centredness[Title/Abstract])) OR (person-centered nursing framework[Title/Abstract])) OR (personalized care[Title/Abstract])) OR (pe-ce[Title/Abstract])) AND (((((((((((((((Nursing Homes[MeSH Terms]) OR (Nursing Homes[Title/Abstract])) OR (homes for the aged[MeSH Terms])) OR (homes for the aged[Title/Abstract])) OR (Residential Facilities[MeSH Terms])) OR (Residential Facilities[Title/Abstract])) OR (Long-Term Care[MeSH Terms])) OR (Long-Term Care[Title/Abstract])) OR («homes for the aged»[Title/Abstract])) OR (Long Term Care Patients[Title/Abstract])) OR (municipal home*[Title/Abstract])) OR (Nursing home*[Title/Abstract])) OR (Residential Facilit*[Title/Abstract])) OR (Residental care[Title/Abstract])) OR («Long-term care facilities for older people»[Title/Abstract]))** |

| 1. Leaders/leadership AND attitudes AND person-centred AND implementation etc. (S1 AND S2 AND S3 AND S5)   645 hits |
| --- |
| **(((((((((((Institutional Management Teams[MeSH Terms]) OR (Leadership[MeSH Terms])) OR (Influentials[MeSH Terms])) OR (Personnel Management[MeSH Terms])) OR (leader*[Title/Abstract])) OR (situational leadership[Title/Abstract])) OR ("Nursing Homes/organization and administration"[Mesh])) OR ("Institutional Management Teams/organization and administration"[Mesh])) OR ("Personnel Management/organization and administration"[Mesh])) AND (((((((((Attitude of Health Personell)[MeSH Terms]) OR (Attitude of Health Personell[Title/Abstract]))) OR (Attitude[MeSH Terms])) OR (Attitude[Title/Abstract])) OR (Work Engagement[MeSH Terms])) OR (Work Engagement[Title/Abstract])) OR ("staff attitude*"[Title/Abstract])) OR ("Attitude of Health Personnel/organization and administration"[Mesh])) OR ("Attitude/organization and administration"[Mesh]))) AND (((((((((((((((((((Patient-Centered Care[MeSH Terms]) OR (Patient-Centered Care[Title/Abstract])) OR (Patient Care Planning[MeSH Terms])) OR (Patient Care Planning[Title/Abstract])) OR ("Patient Care Planning/organization and administration"[Mesh])) OR ("Patient-Centered Care/organization and administration"[Mesh])) OR (person-centered practice[Title/Abstract])) OR (person-centred care[Title/Abstract])) OR (person-centred healthcare[Title/Abstract])) OR (patient-centered care[Title/Abstract])) OR (people centered care[Title/Abstract])) OR (person-centredness[Title/Abstract])) OR (personhood[Title/Abstract])) OR (individualized care[Title/Abstract])) OR (person-directed care planning[Title/Abstract])) OR (person centredness[Title/Abstract])) OR (person-centered nursing framework[Title/Abstract])) OR (personalized care[Title/Abstract])) OR (pe-ce[Title/Abstract]))) AND (((((((((((((((((((Health Plan Implementation[MeSH Terms]) OR ("Health Plan Implementation/organization and administration"[Mesh])) OR (Health Plan Implementation[Title/Abstract])) OR (Quality Improvement[MeSH Terms])) OR ("Quality Improvement/organization and administration"[Mesh])) OR (Quality Improvement[Title/Abstract])) OR (Organizational Culture[MeSH Terms])) OR (Organizational Culture[Title/Abstract])) OR («health plan implementation»[Title/Abstract])) OR ("culture change"[Title/Abstract])) OR (framework[Title/Abstract])) OR (quality improvement[Title/Abstract])) OR (organizational change[Title/Abstract])) OR ("program implementation¨[Title/Abstract])) OR (Implementation[Title/Abstract])) OR (perspective[Title/Abstract])) OR (innovation[Title/Abstract])) OR (experience[Title/Abstract])) OR (involvement[Title/Abstract]))** |
